# A Pilot Electroencephalography Study of the Effect of CT1812 Treatment on Synaptic Activity in Patients with Mild to Moderate Alzheimer’s Disease

**DOI:** 10.14283/jpad.2024.154

**Published:** 2024-08-07

**Authors:** E. Vijverberg, W. de Haan, E. Scheijbeler, M. E. Hamby, S. Catalano, P. Scheltens, M. Grundman, Anthony O. Caggiano

**Affiliations:** 1 Alzheimer Center Amsterdam, Neurology, Vrije Universiteit Amsterdam, Amsterdam UMC location VUmc, Amsterdam, The Netherlands; 2 Cognition Therapeutics, Inc., 2500 Westchester Avenue, Purchase, NY 10577 USA; 3 Capsida Biotherapeutics, Thousand Oaks, CA USA; 4 Global R&D Partners, LLC, San Diego, California USA; 5 Department of Neurosciences, University of California, San Diego, USA

**Keywords:** Aβ oligomer, Alzheimer’s disease, CT1812, quantitative electroencephalogram, sigma-2 receptor modulator

## Abstract

**Background:**

CT1812 is a first-in-class, sigma-2 receptor ligand, that prevents and displaces binding of amyloid beta (Aβ) oligomers. Normalization of quantitative electroencephalography (qEEG) markers suggests that CT1812 protects synapses from Aβ oligomer toxicity.

**Objectives:**

Evaluate CT1812 impact on synaptic function using qEEG measurements.

**Design:**

Phase 2, randomized, double-blind, placebo-controlled, 4-week crossover study.

**Setting:**

VU University Medical Center and Brain Research Center Amsterdam, The Netherlands.

**Participants:**

Adults with mild or moderate Alzheimer’s disease (AD).

**Intervention:**

A daily 300 mg dose of CT1812 or placebo for 4 weeks.

**Measurements:**

A resting-state, eyes closed qEEG assessment occurred on Day 1 and on Day 29 of Treatment Periods 1 and 2, and at follow-up. The primary endpoint was global relative theta power (4–8 Hz), along with secondary EEG measures including global alpha corrected Amplitude Envelope Correlation (AEC-c). Cognitive and functional assessments, fluid biomarkers, and safety and tolerability were assessed.

**Results:**

16 patients were randomized, and 15 completed. A non-significant (p=0.123) but consistent reduction occurred in global relative theta power and in relative theta power in frontal, temporal, parietal, occipital and central (p<0.006) brain regions with CT1812. A nominally significant (p=0.034) improvement was observed in global alpha AEC-c. Adverse events occurred in 11 patients with CT1812 and 6 with placebo -most commonly nausea, diarrhea, and procedural headache. No severe or serious AEs, deaths or discontinuations were reported.

**Conclusion:**

CT1812 improved established EEG markers of spontaneous brain activity (spectral power, functional connectivity) in patients with mild-to-moderate AD, suggesting improved neuronal/synaptic function within a 4-week timespan.

**Electronic Supplementary Material:**

Supplementary material is available in the online version of this article at 10.14283/jpad.2024.154.

## Introduction

Synaptic and neuronal dysfunction and loss caused by age-dependent accumulation of synaptotoxic amyloid-beta 42 (Aβ42) oligomers may underlie cognitive decline that is typical in patients with Alzheimer’s disease (AD) (1). Accumulation of Aβ42 leads to formation of oligomers, which bind to receptor sites on the surface of neuronal synapses (2, 3). After binding, these oligomers alter membrane trafficking rate and reduce surface expression of neuronal receptors that are critical for maintaining synaptic plasticity (4, 5). This leads to failure of long-term potentiation, spine loss in neurons, and impaired cognitive performance that progresses throughout the course of AD (6–8). The sigma-2 receptor (S2R), which is encoded by transmembrane protein 97 (TMEM97), is found in different cell types including neurons. S2R regulates cell function through the progesterone receptor membrane component 1 (PGRMC1) receptor and mediates binding of Aβ oligomers to neurons in AD (2, 3).

CT1812 is a novel, first-in-class, highly brain penetrant sigma-2 receptor ligand, currently in clinical development for dementia (Alzheimer’s disease and dementia with Lewy bodies). CT1812 has demonstrated disease-modifying properties in animal models and is believed to function by preventing binding and displacing Aβ42 oligomers from receptors on brain cells (8–11). In cultured rat neurons, CT1812 antagonizes binding of Aβ42 oligomers, prevents Aβ oligomer-induced membrane trafficking changes and restores synapse protein expression (8). Chronic treatment with CT1812 restores aged AD model transgenic mouse cognitive performance to normal using multiple tests (8). Likewise, CT1812 may restore neuronal plasticity compromised by Aβ oligomers and may improve cognitive function in patients throughout the course of AD.

Various techniques can be used to study brain activity and functional connectivity, but quantitative electroencephalography (qEEG) is widely available in the hospital setting and is non-invasive, cheap, and reliable. qEEG is a neurophysiological modality that measures electrical brain activity with a high temporal resolution, providing a relatively direct measure of synaptic function. Neuronal dysfunction caused by cerebral pathology including the deposition of toxic Aβ oligomers in AD, can be detected by qEEG (12). Measures expressing local brain activity are used in the diagnostic evaluation of patients with AD in clinical care where the qEEG shows robust changes reflecting abnormalities of brain activity that may reflect disease stage (13–18).

In the prodromal phase of AD, qEEG can be normal. With progression of the disease, there is a gradual, diffuse slowing of oscillatory activity, reflected by changes of spectral power in conventional frequency bands. First, slow (theta; 4–8 Hz) activity increases and fast (beta, 13–30 Hz) activity decreases, followed by slowing and diminished reactivity of mid-range (alpha, 8–13 Hz) activity. Finally, very slow (delta, 0.5–4 Hz) power increases (15). In amyloid-positive patients who have no objective cognitive disturbances, global theta power is a predictive marker of future cognitive decline (15). Quantitative EEG measures previously have been used successfully as outcome measure in AD trials, both in the older cholinesterase inhibitor literature (19–21) and in more recent studies with different therapeutic targets, including amyloid-beta (17, 18, 22). Since global relative theta power is the most powerful early-phase diagnostic qEEG marker in AD patients (23), it was included in this study as the primary efficacy outcome measure. Several other measures including relative power in the alpha and beta bands, the global theta/alpha power ratio, posterior peak frequency and amplitude-based functional connectivity in the alpha band were also assessed (15, 24). Functional connectivity findings including reduced AECc alpha connectivity in AD were evaluated (25).

The hypothesis was tested that treatment with CT1812 can normalize qEEG patterns by improving functional connectivity and decreasing theta power relative to placebo, and that this may be an indication that CT1812 protects synapses from the toxicity of Aβ oligomers (26, 27). Normalization of the EEG means a decrease (or stabilization) of theta and increase in alpha power and functional connectivity (28). Safety was assessed and proof of mechanism of CT1812 was demonstrated in early clinical studies in healthy volunteers and patients with AD (8, 9, 29–31).

The objectives of this study were to evaluate the effect of CT1812 in restoring synaptic function in participants with mild to moderate AD using qEEG measurements and to confirm the safety and tolerability of CT1812 over a 29 day treatment course.

## Methods

The study was conducted in accordance with the International Conference on Harmonization Tripartite Guideline for Good Clinical Practice, and the Guidelines of the Declaration of Helsinki. The study protocol and informed consent form were reviewed and approved by an institutional review board. All participants and their caregivers provided written informed consent prior to any study procedures.

### Study Design

This was a single-center (VU University Medical Center and Brain Research Center, Amsterdam, The Netherlands), randomized, double-blind, placebo-controlled, 29-day, 2-period, crossover, Phase 2 study in adults with mild to moderate AD (EudraCT: 2019-003552-36; clinicaltrials.gov: NCT04735536). Screening procedures occurred on Days -42 to -1. Eligible participants returned to the study site at the baseline visit and were randomly assigned in Treatment Period 1 to either CT1812 or placebo daily for 4 weeks, followed by a 2-week washout period. In Treatment Period 2, patients received the alternate treatment with placebo or CT1812, and returned to the study site 12 days after the final dose of study treatment for follow-up safety and laboratory assessments. The total duration of participation in the study, including the screening period, was up to 126 days.

### Patient Selection

Women and men, aged 50 to 85 years, inclusive, were eligible for inclusion if they had a diagnosis of mild to moderate probable Alzheimer’s disease dementia according to Alzheimer’s Association-National Institute on Aging (AA-NIA) 2018 criteria (32) and at least a 6-month documented history of decline in cognitive function. Participants had to use appropriate contraceptive measures during and after the trial.

To confirm the diagnosis of AD, cerebrospinal fluid (CSF) sample had to meet Aβ42 and p-tau-181 criteria: CSF Aβ42 <1000 pg/mL AND CSF p-tau 181 >19 pg/mL; OR CSF Aβ1–42 <1000 pg/mL AND p-tau-181 / Aβ42 ratio >0.020; OR CSF p-tau 181 >19 pg/mL AND p-tau-181 / Aβ1–42 ratio >0.020, as measured via an Elecsys assay. Historical CSF results were considered provided the results were consistent with the CSF thresholds required for inclusion and following discussion with the medical monitor; however, a lumbar puncture was still required as part of screening procedures.

Magnetic resonance neuroimaging (MRI) findings consistent with the clinical diagnosis of AD and without findings of significant exclusionary abnormalities were required. A historical MRI up to 1 year prior to screening was allowed as long as no clinical neurologic events occurred during this interval, which suggested a change in the MRI scan. In addition, a score of 18 to 26 on the mini-mental status examination (MMSE) (33), and no active depression and a Geriatric Depression Scale (GDS) (34) score ≤6 were required.

Participants were required to have a caregiver/study partner who had contact with the study participant for a sufficient number of hours per week to provide informative responses on study assessments, oversee administration of the study drug, and who was willing and able to participate in all study site visits and study assessments. Participants had to be living at home or in the community (assisted living acceptable) with no known history of difficulty swallowing capsules; on stable pharmacological treatment with acetylcholinesterase inhibitors or memantine for any other chronic conditions for at least 30 days prior to screening; willing to undergo apolipoprotein E (APOE) genotyping; and be generally healthy with mobility (ambulatory or ambulatory-aided, i.e., walker or cane), vision, and hearing (hearing aid permissible) sufficient for compliance with testing procedures. Participants were excluded for anything that might interfere with the conduct of the study. Detailed Inclusion/Exclusion criteria and prohibited medications are listed in Supplementary Materials.

### Study Assessments

At screening, the MMSE and GDS were administered, MRI imaging and a lumbar puncture was performed, and apolipoprotein (ApoE) status was determined. Safety assessments included physical examination, vital signs (blood pressure, heart rate, body temperature), clinical laboratory testing (chemistry, hematology, urinalysis), 12-lead electrocardiogram (ECG), and adverse events. Serum concentrations of CT1812 were assessed at screening, 1 hour predose on Days 1, 8, 15, 22, 29, 44, 51, 58, 65 and 72 and 2 hours postdose on Days 1, 29, 44, and 72. CSF concentrations of CT1812 were measured at screening and 24 hours after the dose on Days 29 and 72. To assess target engagement and disease modification, plasma and CSF concentrations of Aβ42 and Aβ40 monomers, phospho-tau-217, phospho-tau-181, neurofilament light (NFL), glial fibrillary acidic protein (ng/L) were measured, and the Aβ42/40 ratio calculated. In addition, CSF concentrations of chitinase-3-like protein 1 (YLK-40, mg/L), neurogranin, neuronal pentraxin 2 (ng/L), soluble triglyceride receptor expressed on myeloid cells 2 (TREM2, ng/L), synaptosome associated protein 25 (SNAP25, ng/L); total tau (mg/dL), and vesicle-associated membrane protein 2 (VAMP2, ng/L) were measured.

Quantitative EEGs were recorded on OSG digital equipment (BrainRT™; OSG BV, Rumst, Belgium) with Ag-Cl electrodes. An EEG assessment was conducted during a 15-minute task-free session with closed eyes on Day 1 and on Day 29 of Treatment Period 1, at Days 44 and 72 (Day 1 and Day 29 of Treatment Period 2), and at the 84 day follow-up. Twenty-one channels of the standard 10–20 system were recorded: Fp2 / Fp1, F8 / F7, F4/ F3, A2 / A1, T4 / T3, C4 / C3, T6 / T5, P4 / P3, O2 / O1, Fz, Cz, Pz, an ECG– and a respiration channel. Raw data were recorded using a montage with Fz as the common reference electrode. Electrode impedance was checked before and after the recording and was kept below 5 Kohm. Filter settings were high pass 0.16 Hz, low pass 70 Hz, and no notch filter. A sample frequency of 512 Hz and analog-to-digital conversion precision of 12 bit was used. Care was taken by an experienced EEG technician, who was blinded to treatment condition during the recording, to keep participants in an eyes-closed but vigilant condition, and to minimize any other artifacts. Five 8.2 s epochs of eyes-closed artifact-free data (containing no eye blinks, muscle artifact, slow eye movements, or EKG-artifacts) were selected from each EEG recording based on visual inspection of the data by a trained EEG technician.

The primary qEEG endpoint was global relative theta power (4–8 Hz), the fraction of total oscillatory activity in all cortical regions accounted for by theta wave frequency. Secondary qEEG measures included global relative alpha (8–13 Hz) and global relative beta (13–30 Hz) power, theta/alpha power ratio, posterior peak frequency (i.e., the dominant frequency of the power spectrum between 4–13 Hz in the parieto-occipital region), and global functional connectivity assessed with Amplitude Envelope Correlation (AEC-c) in the alpha band (27, 35–37). Details provided in the Supplementary Material.

Cognition and global functioning were assessed at baseline and Days 29, 44, and 72 with the Alzheimer’s disease assessment scale–cognition subscale (ADAS-Cog-14) (38) and Alzheimer’s Disease Cooperative Study-Clinical Global Impression of Change (ADCS-CGIC) (39). The Columbia Suicide Severity Rating Scale (C-SSRS) (40) was administered at screening and baseline and Days 29, 44, and 72. Plasma drug concentrations were measured predose on Days 1, 8, 15, 22, and 29 and postdose on Days 1 and 29. CSF concentrations were measured predose on Day 29.

### Statistical Analysis

For the primary qEEG endpoint, global relative theta power, the assumed within-subject standard deviation (SD) was equal to 3%. Based on the use of a two-sided one-sample (within-subject) comparison between CT1812 and placebo at the alpha=0.05 level of significance, a sample size of 16 participants provided 90% power to detect a mean difference between treatments of 2.5%. Standard non-parametric statistical analyses were used to examine the relationships between CT1812 dose and PK parameters. Pharmacodynamics markers in CSF and CT1812 concentrations in serum and CSF were summarized using descriptive statistics.

## Results

Of 34 patients who were screened, 16 were randomized to study treatment, and 15 completed the study. One patient discontinued from the crossover CT1812/placebo group for withdrawal of consent (death in the family). At baseline, mean (SD) age was 66.4 (7.9) years, 50% were female, and mean (SD) time since diagnosis of AD was 1.14 (1.0) years (Table 1). At baseline, mean (SD) MMSE was 21.1 (2.4), ADAS-Cog14 was 30.2 (6.9), and Amsterdam IADL was 52.6 (5.1). All participants had an AA-NIA diagnosis of mild AD, and 6 of 16 patients and 5 of 16 patients were hetero- or homozygous positive for ApoE ε4.

**Table 1:** Baseline characteristics (safety population)

**Characteristics**	**Placebo/CT1812 (n=8)**	**CT1812/Placebo (n=8)**
Age, years ^a^	65.6 ± 6.2	67.3 ± 9.7
Age range, years	54–74	51–81
Female, n (%)	4 (50.0)	4 (50.0)
White, non-Hispanic, n (%)	8 (100)	8 (100)
Body mass index, kg/m^2^ ^a^	26.1 ± 5.4	25.7 ± 2.7
Time since diagnosis, years ^a^	1.4 ± 1.2	0.9 ± 0.6
MMSE total score, screening ^a^	21.4 ± 1.7	20.8 ± 3.0
ADAS-Cog14 total score, baseline ^a^	30.6 ± 7.6	29.8 ± 6.5
Amsterdam IADL ^a^	52.4 ± 5.5	52.9 ± 5.0
ApoE status, genotypes, n (%)		
ApoE e3/e3	1 (12.5)	4 (50.0)
ApoE e3/e4	4 (50.0)	2 (25.0)
ApoE e4/e4	3 (37.5)	2 (25.0)
Screening APoE status - rs429358, n (%)		
CC	3 (37.5)	2 (25.0)
TC	4 (50.0)	2 (25.0)
TT	1 (12.5)	4 (50.0)
Screening APoE status - rs7412, n (%)		
CC	8 (100)	8 (100)

a. Mean ± standard deviation

### Quantitative Electroencephalogram

**Figure 1:**
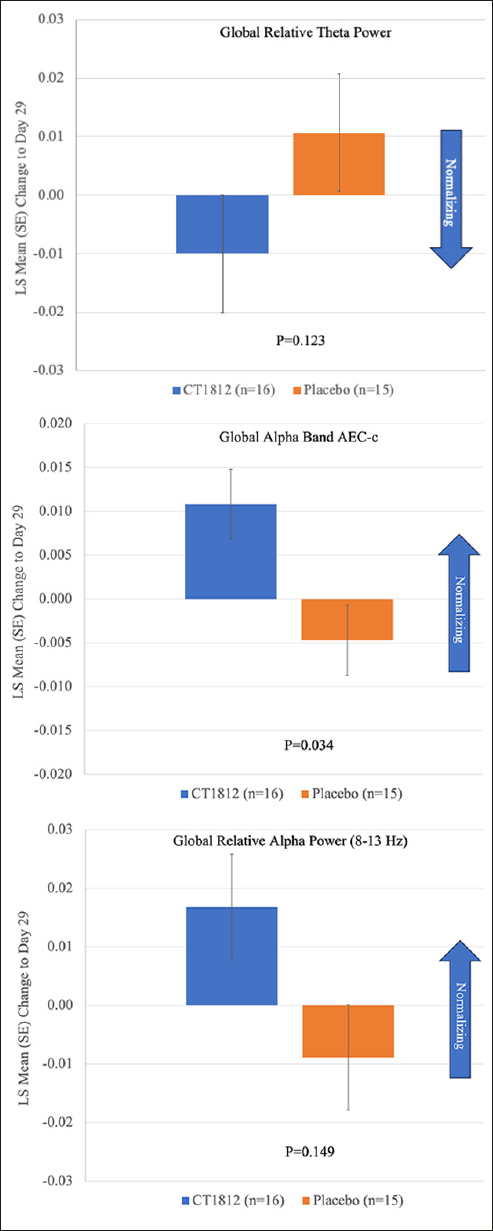
Mean change from baseline for Global Relative Theta Power, Global Alpha AEC-c, and Global Relative Alpha Power

**Figure 2:**
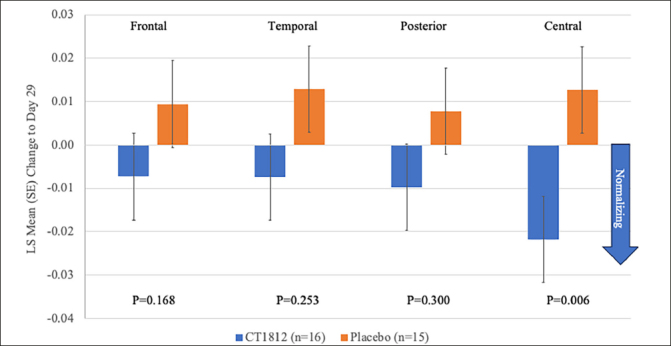
Mean Change from Baseline for Regional Relative Theta Power

Following treatment with CT1812 for 4 weeks, a nonsignificant reduction (LS mean [standard error] difference: −0.021 [0.013]; p=0.123) was observed in global relative theta power (Figure 1, Table 2). A consistent trend for decreases in relative theta power also were observed in frontal, temporal, posterior (parietal and occipital), and central brain regions (Figure 2, Table 2). The change observed in the central region was statistically significant (LS mean [SE] difference: −0.035 [0.011]; p<0.006). A nominally significant (LS mean [SE] difference: 0.016 [0.007]; p=0.034) improvement in global alpha AEC-c was observed with CT1812 (Figure 1, Table 2). A numerically favorable treatment difference (LS mean [SE] difference: 0.026 [0.017]; p=0.149) was observed for increases in global relative alpha power with CT1812 (Figure 1, Table 2).

Plasma and CSF concentrations of exploratory pre-specified biomarkers were relatively unchanged from baseline to Day 29 (Supplemental Tables 1 and 2). Mean (SD) ADAS-Cog-14 scores were 30.1 (7.2) and 29.9 (7.6) at baseline with placebo and CT1812 and mean (SD) change to Day 29 was −0.8 (5.2) with placebo and −0.7 (6.3) with CT1812 (LS mean difference 0.2, 95% CI: −2.7, 3.0, p=0.894). No significant differences were observed between CT1812 and placebo for cognitive or functional outcomes (Supplemental Table 3). At Day 29, mean (SD) ADCS-CGI-I scores were 4.0 (0.76) with placebo and 4.0 (0.73) with CT1812; all 16 patients with CT1812 had minimal or no change as did 14 of 15 patients with placebo. No differences between groups were apparent for Amsterdam IADL and the NTI Battery. Mean (SD) predose plasma concentrations of CT1812 were 25.5 (25.0), 14.9 (7.8), 13.8 (8.0), and 14.1 (9.0) ng/mL on Days 8, 15, 22, and 29, respectively. On Day 29, mean (SD) predose CSF concentration of CT1812 was 0.41 (0.25) ng/mL.

### Safety/Tolerability

Mild or moderate treatment-emergent adverse events (TEAEs) occurred in 11 patients with CT1812 and 6 with placebo (Table 3). The most common AEs were nausea, diarrhea, hematoma, and procedural headache. No severe or serious AEs were reported, and no deaths or discontinuations due to TEAEs were reported. Six TEAEs (diarrhea, nausea, and vomiting with placebo; diarrhea, nausea, and hepatic enzyme increased with CT1812) were judged to be treatment-related. One patient experienced an increase in alanine aminotransferase levels that was twice the upper limit of normal and reported as a TEAE. The TEAE was of mild severity and possibly related to CT1812, but treatment was continued.

**Table 2:** Quantitative EEG results for primary and exploratory endpoints (safety population)

	**Placebo (n=15)**		**CT1812 (n=16)**	
	**Observed Value**	**Change from Period Day 1**	**Observed Value**	**Change from Period Day 1**
Primary Endpoint				
*Global Relative Theta Power (uV2/Hz)*				
Mean (SD)	0.228 (0.079)	0.010 (0.032)	0.197 (0.076)	−0.01 (0.043)
LS mean (SE)		0.011 (0.011)		−0.01 (0.010)
LS mean difference (SE)				−0.021 (0.013)
p-value				0.123
Exploratory Endpoints				
*Global Alpha AEC-c*				
Mean (SD)	0.528 (0.019)	−0.005 (0.013)	0.537 (0.024)	0.011 (0.018)
LS mean (SE)		−0.005 (0.004)		0.011 (0.004)
LS mean difference (SE)				0.016 (0.007)
p-value				0.034
*Global Relative Alpha Power (uV2/Hz)*				
Mean (SD)	0.197 (0.085)	−0.008 (0.035)	0.219 (0.101)	0.017 (0.037)
LS mean (SE)		−0.009 (0.010)		0.017 (0.009)
LS mean difference (SE)				0.026 (0.018)
p-value				0.149
*Regional Relative Theta Power – Central (uV2/Hz)*				
Mean (SD)	0.246 (0.093)	0.011 (0.039)	0.208 (0.089)	−0.022 (0.048)
LS mean (SE)		0.013 (0.011)		−0.022 (0.011)
LS mean difference (SE)				−0.035 (0.011)
p-value				0.006
*Regional Relative Theta Power – Frontal (uV2/Hz)*				
Mean (SD)	0.202 (0.070)	0.010 (0.034)	0.173 (0.069)	−0.007 (0.042)
LS mean (SE)		0.009 (0.010)		−0.073 (0.010)
LS mean difference (SE)				−0.017 (0.011)
p-value				0.168
*Regional Relative Theta Power – Posterior (uV2/Hz)*				
Mean (SD)	0.225 (0.080)	0.008 (0.039)	0.190 (0.082)	−0.010 (0.043)
LS mean (SE)		0.008 (0.011)		−0.010 (0.011)
LS mean difference (SE)				−0.018 (0.016)
p-value				0.300
*Regional Relative Theta Power - Temporal (uV2/Hz)*				
Mean (SD)	0.250 (0.093)	0.013 (0.040)	0.226 (0.082)	−0.007 (0.049)
LS mean (SE)		0.013 (0.012)		−0.007 (0.012)
LS mean difference (SE)				−0.020 (0.017)
p-value				0.253

Least square (LS) mean, standard errors (SE), and p-value are estimated using a linear mixed model with fixed effects for treatment group (CT1812, placebo), sequence, and period, and a random effect for subject within sequence.

**Table 3:** Incidence of treatment-emergent adverse events (TEAE) occurring in more than one patient (safety population)*

	**Number (%) of Patients**	
	**CT1812 (n=16)**	**Placebo (n=15)**
Any TEAE	11 (68.8)	6 (40.0)
Any related TEAE	3 (18.8)	3 (20.0)
Individual TEAEs		
Chronic virus infection	1 (6.3)	1 (6.7)
Diarrhea	1 (6.3)	1 (6.7)
Headache	2 (12.5)	0
Hematoma	2 (12.5)	1 (6.7)
Nausea	2 (12.5)	1 (6.7)
Procedural headache	3 (18.8)	1 (6.7)

* Single TEAEs occurring either group: CT1812 – atrial fibrillation, bone contusion, first-degree burn, hepatic enzyme increased; placebo – first-degree burn, pneumonia, post-procedural contusion, vomiting

No clinically relevant changes were observed from baseline to Day 29 for hematology testing or urinalysis. On the C-SSRS, suicide ideation or suicide behavior were reported in a single patient during treatment.

## Discussion

In this study, qEEG was used to determine the effect of CT1812 on AD pathophysiology in the brain. Normalization would indicate a treatment effect, as this does not happen spontaneously during the disease, and we found a positive effect on oscillatory brain activity after only 4 weeks of CT1812 treatment. Results from this study support improvement in the neurophysiology of AD with CT1812. Positive, consistent trends were observed with CT1812 for the first three ranked outcomes measures (global relative theta power, global alpha AEC-c, and global relative alpha power). A consistent reduction in global and regional theta power indicates a positive trend toward normalizing brain activity with CT1812. Global alpha AEC-c reflects the ability of the brain to communicate and exchange information between brain regions and this ability is reduced in those with AD (25, 27). In this study, global alpha AEC-c was significantly improved with CT1812. In this 29-day study, CT1812 was well tolerated with only mild to moderate AEs, no serious or severe AEs, and no discontinuations or deaths attributable to CT1812.

Our results are consistent with previous trials that used resting-state qEEG outcome measures (17, 25). In this 4-week study, we observed the pre-specified hypothesized changes. We observed a non-significant but consistent trend in the direction of changes that was similar to a previous study with qEEG in patients with mild or moderate AD treated for 5 days with rivastigmine (21). Of note, EEG demonstrated trends for improvement but no differences were detected in CSF and plasma biomarkers over this short treatment course. While these findings might result from a mechanistically distinct effect of CT1812, the favorable effect could be more general to a class of synaptoprotective drugs. Thus, EEG can be considered an important downstream surrogate marker of improvement.

Limitations of this study include a small sample size and a short duration of follow up. It should be noted that this study was intended to further explore the mechanism of action of CT1812. In addition, the design of the study was not adequate for showing improvements in functional and cognitive outcomes and biomarkers of AD. We focused on resting-state data, which is important given previous results, but task data may provide complementary insights. Further, qEEG mainly reflects cortical activity, while in AD, subcortical activity (e.g., hippocampal) also is important. Magnetoencephalography (MEG) can yield more detailed changes, including from deeper regions of the brain, and a single-site study with MEG would be feasible (35).

In conclusion, results from this study showed that resting state qEEG improved after CT1812. Although qEEG is an exploratory endpoint in patients with AD, substantial evidence suggests that qEEG can detect changes in both whole-brain and regional electrical patterns that are impaired in AD. Results from this study suggest that CT1812 has an effect on neurophysiological endpoints, and contribute to the growing body of preclinical and clinical evidence of the benefits of CT1812. Consistent trends for improvement were observed for pre-specified qEEG measures with significant treatment differences noted for global alpha AECc and central relative theta power. Thus, the results from this study provided encouraging preliminary results of an impact of CT1812 on brain activity in patients with mild-to-moderate AD. Larger proof-of-concept studies with CT1812 are ongoing in patients with early AD (START, NCT05531656), mild-to-moderate AD (SHINE, NCT03507790) (41), and dementia with Lewy bodies (SHIMMER, NCT05225415) that will evaluate the potential role of CT1812 as a disease-modifying therapy in patients with AD and other neurodegenerative disorders.

## Supplementary Material

Supplementary material, approximately 41.3 KB.

## Data Availability

*Data sharing statement:* All data for this study are reported in the manuscript and Data Supplement. Additional data may be available from the sponsor upon the written request.
