# Supplementary material for: A Pilot Electroencephalography Study of the Effect of CT1812 Treatment on Synaptic Activity in Patients with Mild to Moderate Alzheimer’s Disease
Source: J Prev Alzheimers Dis. 2024 Aug 7;11(6):1809–17. doi: 10.14283/jpad.2024.154 (PMC11573871; doi:10.14283/jpad.2024.154)
Supplement: Supplementary file 1 — Supplementary material, approximately 41.3 KB. [file 42414_2024_154_MOESM1_ESM.docx]

**Supplementary Material**

**Detailed Inclusion/Exclusion Criteria**

Participants may be included in the study only if they meet all of the following criteria:

1. Women of non-childbearing potential and men, aged 50 to 85 years, inclusive, with a diagnosis of mild to moderate Alzheimer’s disease according to the 2018 NIA-AA criteria and at least a 6-month history of decline in cognitive function documented in the medical record.

i)  Non-childbearing potential for women is defined as postmenopausal (last menses greater than 24 months) or undergone a documented bilateral tubal ligation or hysterectomy. If last menses less than 24 months, a serum follicle stimulating hormone (FSH) value confirming post-menopausal status may be used.

ii)  Male participants who are sexually active with a woman of child-bearing potential must agree to use condoms during the study and for 3 months after last dose. Female partners should also consider using an acceptable means of birth control, though it is not mandatory. Acceptable forms of birth control include abstinence, birth control pills, or any double combination of: intrauterine device (IUD), male or female condom, diaphragm, sponge, and cervical cap.

1. CSF meets CSF abeta 1‐42 (abeta) and p-tau -181 criteria as defined below:

CSF abeta 1‐42 < 1000pg/ml (Elecsys assay) AND CSF p‐tau 181 > 19 pg/ml (Elecsys Assay) OR: CSF abeta 1‐42 < 1000pg/ml (Elecsys assay) AND p‐tau ‐181 / abeta 1‐42 ratio > 0.020

OR: CSF p‐tau 181 > 19 pg/ml (Elcsys Assay) AND p‐tau ‐181 / abeta 1‐42 ratio > 0.020

Historical CSF results will be considered provided the results are consistent with the CSF thresholds required for inclusion and following discussion with the medical monitor; however, an LP is still required as part of screening procedures.

1. Neuroimaging (MRI) consistent with the clinical diagnosis of Alzheimer’s disease and without findings of significant exclusionary abnormalities (see Section 9.3 exclusion criteria no. 4). An historical MRI, up to 1 year prior to screening, may be used as long as there have been no interval clinical neurologic events that may suggest a change in the MRI scan.
2. MMSE 18-26 inclusive.
3. Geriatric Depression Scale (GDS) ≤ 6 with no active depression (see Section 9.3 exclusion criteria no. 6).
4. Formal education of 8 or more years.
5. Participants must have a caregiver/study partner who in the opinion of the site’s Principal Investigator, has contact with the study participant for a sufficient number of hours per week to provide informative responses on the protocol assessments, oversee the administration of study drug, and is willing and able to participate in all study site visits and some study assessments. The caregiver/ study partner must provide written informed consent to participate in the study.
6. Participants living at home or in the community (assisted living acceptable).
7. Participants must have no known history of difficulty swallowing capsules.
8. Stable pharmacological treatment of any other chronic conditions for at least 30 days prior to screening.
9. Must consent to apolipoprotein E (APOE) genotyping.
10. Participants shall be generally healthy with mobility (ambulatory or ambulatory-aided, i.e., walker or cane), vision and hearing (hearing aid permissible) sufficient for compliance with testing procedures.
11. Must be able to complete all screening evaluations.

**Exclusion Criteria**

Participants will be excluded from the study if any of the following conditions apply:

1. Hospitalization (except for planned procedures) or change of chronic concomitant medication within 1 month prior to screening.
2. Participants living in a continuous care nursing facility.
3. Contraindications to the MRI examination for any reason.
4. Screening MRI (or historical MRI, if applicable) of the brain indicative of significant abnormality, including, but not limited to, prior hemorrhage or infarct > 1 cm3, > 3 lacunar infarcts, cerebral contusion, encephalomalacia, aneurysm, vascular malformation, subdural hematoma, hydrocephalus, space-occupying lesion (e.g., abscess or brain tumor such as meningioma).
5. Clinical or laboratory findings consistent with:

a)  Other primary degenerative dementia, (dementia with Lewy bodies, fronto-temporal dementia, Huntington’s disease, Creutzfeldt-Jakob Disease, Down syndrome, etc.).

b)  Other neurodegenerative condition (Parkinson’s disease, amyotrophic lateral sclerosis, etc.).

c)  Seizure disorder.

d)  Other infectious, metabolic or systemic diseases affecting the central nervous system (syphilis, present hypothyroidism, present vitamin B12 or folate deficiency, other laboratory values etc.).

1. A current DSM-V diagnosis of active major depression, schizophrenia or bipolar disorder. Participants with depressive symptoms successfully managed by a stable dose of an antidepressant are allowed entry.
2. Clinically significant, advanced or unstable disease that may interfere with outcome evaluations, such as:

a)  Chronic liver disease, liver function test abnormalities or other signs of hepatic insufficiency (ALT, AST, alkaline phosphatase > 1.5 ULN, lactate dehydrogenase (LDH) > 1.5 x ULN).

b)  Respiratory insufficiency.

c) Renal insufficiency eGFR < 50 mL/min based on the CKD-EPI formula, https://www.mdcalc.com/ckd-epi-equations-glomerular-filtration-rate-gfr

d)  Heart disease (myocardial infarction, unstable angina, heart failure, cardiomyopathy within 6 months before screening).

e)  Bradycardia (< 50/min.) or tachycardia (> 100/min.).

f)  Poorly managed hypertension (systolic > 160 mm Hg and/or diastolic > 95 mm Hg) or hypotension (systolic < 90 mm Hg and/or diastolic < 60 mm Hg).

g)  Uncontrolled diabetes in known diabetics, as defined by hemoglobin A1c (HbA1c) > 7.5.

1. History of cancer within 3 years of screening with the exception of fully excised non- melanoma skin cancers or non-metastatic prostate cancer that has been stable for at least 6 months.
2. Seropositive for human immunodeficiency virus (HIV).
3. History of acute/chronic hepatitis B or C and/or carriers of hepatitis B (seropositive for hepatitis B surface antigen [HbsAg] or anti-hepatitis C [HCV] antibody).
4. Clinically significant abnormalities in screening laboratory tests, including:

a) Hematocrit less than 35% for males and less than 32% for females, absolute neutrophil cell count of <1500/uL (with the exception of a documented history of a chronic benign neutropenia), or platelet cell count of < 120,000/uL; international normalized ratio (INR) > 1.4 or other coagulopathy, confirmed by repeat assessment of:

i) Hematocrit.

ii)  Neutrophil count.

iii)  Platelet count.

1. Disability that may prevent the participant from completing all study requirements (e.g., blindness, deafness, severe language difficulty, etc.).
2. Within 4 weeks of screening visit or during the study, concurrent treatment with antipsychotic agents, antiepileptics, centrally active anti-hypertensive drugs (e.g., clonidine, l-methyl dopa, guanidine, guanfacine, etc.), sedatives, opioids, mood stabilizers (e.g., valproate, lithium); or benzodiazepines, with the following exception:

a) Low dose lorazepam may be used for sedation prior to MRI scan for those participants requiring sedation. At the discretion of the Investigator, 0.5 to 1 mg may be given orally prior to scan with a single repeat dose given if the first dose is ineffective. No more than a total of 2 mg lorazepam may be used for the MRI scan.

1. Any disorder that could interfere with the absorption, distribution, metabolism or excretion of drugs (e.g., small bowel disease, Crohn’s disease, celiac disease, or liver disease).
2. Nootropic drugs except stable AD meds (acetylcholinesterase inhibitors and memantine).
3. Suspected or known drug or alcohol abuse, i.e., more than approximately 60 g alcohol (approximately 1 liter of beer or 0.5 liter of wine) per day.
4. Suspected or known allergy to any components of the study treatments.
5. Enrollment in another investigational study or intake of investigational drug within the previous 30 days or 5 half-lives of the investigational drug, whichever is longer.
6. Intake of drugs or substances potentially involved in clinically significant induction or inhibition of CYP3A4 or P-gp mediated drug interactions with CT1812, within 4 weeks or 5 half-lives of the interacting drug prior to administration of CT1812 and throughout the study. Grapefruit juice should be avoided in the 2 weeks prior to dosing and throughout the study.
7. Exposure to immunomodulators, anti Aβ vaccines, passive immunotherapies for AD (e.g., monoclonal antibodies) within the past 180 days and/or exposure to BACE inhibitors within the past 30 days
8. Anticipated use of nonsteroidal anti-inflammatory drugs (NSAIDs) on more than 14 days from Baseline/Day 1 to Day 182. Contraindication to undergoing an LP including, but not limited to: inability to tolerate an appropriately flexed position for the time necessary to perform an LP; international normalized ratio (INR) > 1.4 or other coagulopathy; platelet count of < 120,000/μL; infection at the desired LP site; taking anti-coagulant medication within 90 days of screening (low-dose aspirin is permitted); degenerative arthritis of the lumbar spine; suspected non-communicating hydrocephalus or intracranial mass; prior history of spinal mass or trauma.
9. Any condition, which in the opinion of the Investigator or the Sponsor, makes the participant unsuitable for inclusion.

Table 1. Summary of Plasma Biomarkers (PD Population)

|  | **Placebo**  **N=15** | **CT1812 (300 mg)**  **N=16** |
| --- | --- | --- |
| **Plasma Biomarkers** | **Change from Baseline** | **Change from Baseline** |
| pTau181 (ng/L) |  |  |
| LS Mean (SE) | 2.90 (1.10) | 0.82 (1.03) |
| LS Mean Difference from Placebo (SE) |  | -2.09 (1.39) |
| 95% CI for LS Mean Difference |  | -5.07, 0.90 |
| p-value |  | 0.156 |
| pTau217 (ng/L) |  |  |
| LS Mean (SE) | 0.07 (0.06) | 0.014 (0.06) |
| LS Mean Difference from Placebo (SE) |  | -0.06 (0.06) |
| 95% CI for LS Mean Difference |  | -0.19, 0.07 |
| p-value |  | 0.325 |
| Aβ 40 (ng/L) |  |  |
| LS Mean (SE) | 1.35 (2.00) | -0.59 (1.91) |
| LS Mean Difference from Placebo (SE) |  | -1.94 (1.89) |
| 95% CI for LS Mean Difference |  | -5.99, 2.12 |
| p-value |  | 0.322 |
| Aβ 42 (ng/L) |  |  |
| LS Mean (SE) | 0.11 (0.20) | -0.004 (0.19) |
| LS Mean Difference from Placebo (SE) |  | -0.11 (0.187) |
| 95% CI for LS Mean Difference |  | -0.51, 0.29 |
| p-value |  | 0.564 |
| Aβ 42/Aβ 40 |  |  |
| LS Mean (SE) | 0.0001 (0.001) | 0.0000 (0.001) |
| LS Mean Difference from Placebo (SE) |  | 0.0000 (0.001) |
| 95% CI for LS Mean Difference |  | -0.003, 0.003 |
| p-value |  | 0.976 |
| NFL (ng/L) |  |  |
| LS Mean (SE) | 1.07 (1.14) | 2.0005 (1.07489) |
| LS Mean Difference from Placebo (SE) |  | 0.9281 (1.32340) |
| 95% CI for LS Mean Difference |  | -1.9103, 3.7665 |
| p-value |  | 0.495 |
| GFAP (ng/L) |  |  |
| LS Mean (SE) | 18.71 (12.91) | 15.49 (12.48) |
| LS Mean Difference from Placebo (SE) |  | -3.21 (10.25) |
| 95% CI for LS Mean Difference |  | -25.20, 18.77 |
| p-value |  | 0.759 |

Aβ amyloid beta peptide, CI = confidence interval, GFAP = glial fibrillary acidic protein, LS = least square, NFL = neurofilament light, pTau = phosphorylated Tau, SE = standard error

Table 2. Summary of CSF Biomarkers (PD Population)

|  | **Placebo**  **N=15** | **CT1812 (300 mg)**  **N=16** |
| --- | --- | --- |
| **CSF Biomarkers** | **Change from Baseline** | **Change from Baseline** |
| Aβ 42 (ng/L) |  |  |
| LS Mean (SE) | 6.7 (20.32) | 15.6 (20.31) |
| LS Mean Difference from Placebo (SE) |  | 8.8 (21.76) |
| 95% CI for LS Mean Difference |  | -37.8, 55.5 |
| p-value |  | 0.691 |
| pTau181 (ng/L) |  |  |
| LS Mean (SE) | 0.05 (2.72) | -0.75 (2.65) |
| LS Mean Difference from Placebo (SE) |  | -0.80 (2.84) |
| 95% CI for LS Mean Difference |  | -6.88, 5.28 |
| p-value |  | 0.781 |
| tTau (ng/L) |  |  |
| LS Mean (SE) | 9.5 (19.42) | 13.2 (18.80) |
| LS Mean Difference from Placebo (SE) |  | 3.7 (22.03) |
| 95% CI for LS Mean Difference |  | -43.5, 51.0 |
| p-value |  | 0.868 |
| Aβ 40 (ng/L) |  |  |
| LS Mean (SE) | 320.9 (406.17) | 245.1 (402.83) |
| LS Mean Difference from Placebo (SE) |  | -75.8 (407.37) |
| 95% CI for LS Mean Difference |  | -949.6, 797.9 |
| p-value |  | 0.855 |
| Aβ 42/Aβ 40 |  |  |
| LS Mean (SE) | -0.0004 (0.0006) | 0.0004 (0.0006) |
| LS Mean Difference from Placebo (SE) |  | 0.0008 (0.0007) |
| 95% CI for LS Mean Difference |  | -0.0006, 0.002 |
| p-value |  | 0.244 |
| pTau217 (ng/L) |  |  |
| LS Mean (SE) | -0.85 (2.11) | -0.43 (2.04) |
| LS Mean Difference from Placebo (SE) |  | 0.42 (2.25) |
| 95% CI for LS Mean Difference |  | -4.41, 5.25 |
| p-value |  | 0.855 |
| NFL (ng/L) |  |  |
| LS Mean (SE) | -1.51 (43.59) | -56.92 (41.73) |
| LS Mean Difference from Placebo (SE) |  | -55.41 (48.74) |
| 95% CI for LS Mean Difference |  | -159.94, 49.15 |
| p-value |  | 0.275 |
| Neurogranin (ng/L) |  |  |
| LS Mean (SE) | -15.26 (15.41) | -1.36 (14.05) |
| LS Mean Difference from Placebo (SE) |  | 13.90 (15.09) |
| 95% CI for LS Mean Difference |  | -18.70, 46.50 |
| p-value |  | 0.374 |
| SNAP-25 (ng/L) |  |  |
| LS Mean (SE) | 0.67 (3.49) | 1.48 (3.38) |
| LS Mean Difference from Placebo (SE) |  | 0.81 (3.71) |
| 95% CI for LS Mean Difference |  | -7.15, 8.77 |
| p-value |  | 0.831 |
| GFAP (ng/L) |  |  |
| LS Mean (SE) | -196.53 (1023.57) | 180.13 (978.32) |
| LS Mean Difference from Placebo (SE) |  | 376.66 (1102.53) |
| 95% CI for LS Mean Difference |  | -1988.02, 2741.34 |
| p-value |  | 0.738 |
| YKL-40 (mg/L) |  |  |
| LS Mean (SE) | 0.62 (28.96) | 24.09 (28.72) |
| LS Mean Difference from Placebo (SE) |  | 23.47 (24.13) |
| 95% CI for LS Mean Difference |  | -28.65, 75.60 |
| p-value |  | 0.348 |
| sTREM2 (ng/L) |  |  |
| LS Mean (SE) | -133.6 (109.90) | -17.8 (107.29) |
| LS Mean Difference from Placebo (SE) |  | 115.8 (183.24) |
| 95% CI for LS Mean Difference |  | -280.0, 511.7 |
| p-value |  | 0.538 |
| NXTP2 (ng/L) |  |  |
| LS Mean (SE) | 6.75 (51.46) | 14.27 (48.83) |
| LS Mean Difference from Placebo (SE) |  | 7.52 (44.82) |
| 95% CI for LS Mean Difference |  | -89.32, 104.35 |
| p-value |  | 0.869 |
| VAMP2 (ng/L) |  |  |
| LS Mean (SE) | -12.63 (17.66) | -7.34 (16.749) |
| LS Mean Difference from Placebo (SE) |  | 5.29 (15.39) |
| 95% CI for LS Mean Difference |  | -27.96, 38.53 |
| p-value |  | 0.737 |

Aβ= amyloid beta peptide, CI= confidence intervals, CSF= cerebrospinal fluid, GFAP= glial fibrillary acidic protein, NFL= neurofilament light, NXTP2= neuronal pentraxin 2, pTau= phosphorylated tau, SE= standard error, SNAP‑25= synaptosome associated protein 25, sTREM2= soluble triggering receptor expressed on myeloid cells 2, tTau= total tau, VAMP2= vesicle-associated membrane protein 2, YKL‑40= chitinase-3-like protein 1.

Supplemental Table 3. Functional and cognitive outcomes at Day 29

|  | Placebo (n=15) | | | CT1812 (n=16) | | |  |  |
| --- | --- | --- | --- | --- | --- | --- | --- | --- |
|  | Mean (SD) Baseline | Mean (SD) Day 29 | LS Mean (SE) Change (95% CI) | Mean (SD) Baseline | Mean (SD) Day 29 | LS Mean (SE) Change (95% CI) | LS Mean (SE) Difference vs. Placebo (95% CI) | p-value vs. placebo |
|  |  |  |  |  |  |  |  |  |
| **ADAS-Cog-14** | 30.1 (7.2) | 29.3 (10.1) | -0.8 (5.2) | 29.9 (7.6) | 29.3 (8.8) | -0.7 (6.3) | 0.2 (1.3)  -2.7, 3.0 | 0.894 |
| **Amsterdam IADL Questionnaire** | 52.0 (5.1) | 53.6 (7.2) | 1.6 (3.5) | 54.8 (7.1) | 53.1 (7.0) | -1.7 (4.8) | 3.5 (1.9)  -7.5, 0.5 | 0.083 |
| **NTB** | 13.7 (7.7) | 14.1 (7.5) | 0.3 (5.2) | 11.6 (5.9) | 12.0 (5.2) | 0.4 (3.6 | -0.1 (1.5)  -3.2, 3.0 | 0.936 |

ADAS-Cog-14 = Alzheimer’s disease assessment scale–cognition subscale; IADL = Instrumental Activities of Daily Living Questionnaire (A-IADL-Q); NTB = Neuropsychological Test Battery – Total Generated Words; SD = standard deviation;

LS means, standard errors (SE) and p-value are estimated using a linear mixed model with fixed effects for treatment group (CT1812, placebo), sequence, and period, and a random effect for subject within sequence.
